# Existence of nontrivial weak solutions for a quasilinear Choquard equation

**DOI:** 10.1186/s13660-018-1632-z

**Published:** 2018-02-17

**Authors:** Jongrak Lee, Jae-Myoung Kim, Jung-Hyun Bae, Kisoeb Park

**Affiliations:** 1 Institute of Mathematical Sciences, Ewha Womans University, Seoul, Republic of Korea; 2 Department of Mathematics, Seoul National University, Seoul, Republic of Korea; 3 Department of Mathematics, Sungkyunkwan University, Suwon, Republic of Korea; 4 Department of Mathematics, Incheon National University, Incheon, Republic of Korea

**Keywords:** 35J60, 35J92, 35A15, Weak solutions, Variational method, Choquard equation

## Abstract

We are concerned with the following quasilinear Choquard equation:

$$ -\Delta_{p} u+V(x)|u|^{p-2}u=\lambda\bigl(I_{\alpha} \ast F(u)\bigr)f(u) \quad \text{in } \mathbb {R}^{N}, \qquad F(t)= \int_{0}^{t}f(s) \,ds, $$

where $1< p<\infty$, $\Delta_{p} u=\nabla\cdot(|\nabla u|^{p-2}\nabla u)$ is the *p*-Laplacian operator, the potential function $V:\mathbb {R}^{N}\to(0,\infty)$ is continuous and $F \in C^{1}(\mathbb {R}, \mathbb {R})$. Here, $I_{\alpha}: {\mathbb {R}}^{N}\rightarrow {\mathbb {R}}$ is the Riesz potential of order $\alpha\in(0,p)$. We study the existence of weak solutions for the problem above via the mountain pass theorem and the fountain theorem. Furthermore, we address the behavior of weak solutions to the problem near the origin under suitable assumptions for the nonlinear term *f*.

## Introduction

We are concerned with the following quasilinear Choquard equation:

P $$ -\Delta_{p} u+V(x)|u|^{p-2}u=\lambda \bigl(I_{\alpha}\ast F(u)\bigr)f(u)\quad \text{in } \mathbb {R}^{N}, $$

where $1< p< N$, $\Delta_{p} u=\nabla\cdot(|\nabla u|^{p-2}\nabla u)$ is the *p*-Laplacian operator, the potential function $V:\mathbb {R}^{N}\to(0,\infty)$ is continuous and $F \in C^{1}(\mathbb {R}, \mathbb {R})$ with $F(t)=\int_{0}^{t}f(s) \,ds$. Here, $I_{\alpha}: {\mathbb {R}}^{N}\rightarrow {\mathbb {R}}$ is the Riesz potential of order $\alpha\in(0,p)$ on the Euclidean space ${\mathbb {R}}^{N}$ of dimension $N \geq1$, defined for each $x\in {\mathbb {R}}^{N}\setminus\{0\}$ by

$$I_{\alpha}(x)=\frac{\Gamma(\frac{N-\alpha}{2})}{\Gamma(\frac {\alpha}{2})\pi^{\frac{N}{2}}2^{\alpha}|x|^{N-\alpha}}, $$

where $\Gamma(\cdot)$ stands for a standard Gamma function. The Choquard equation was also introduced by Choquard in 1976 in the modeling of a one-component plasma [1]. It seems to originate from the Frohlich and Pekarí’s model of the polaron, which is a quasiparticle used in condensed matter physics to understand the interactions between electrons and atoms in a solid material [2, 3]. This equation is also known as the Schrödinger–Newton equation in models coupling the Schrödinger equation of quantum physics together with relativistic or nonrelativistic Newtonian gravity [4, 5]. Thus, they have become very significant in physics (see [6] for a review paper).

For this reason, many researchers have extensively studied the Choquard type equation in various ways; see [7–18] and the references therein.

Recently, the authors [19] dealt with the existence of positive solutions to the problem (P) on the whole space $\mathbb{R}^{N}$, based on the assumption that the nonlinearity *f* satisfies the following Ambrosetti–Rabinowitz superlinear condition [20], which is commonly called the (AR)-condition:

$$0< \zeta F(t)\le\theta f(t)t \quad \mbox{for } t > 0 \mbox{ and some } \theta\in \biggl(0, \frac{1}{2}\biggr), $$

where $F(t)=\int_{0}^{t}f(s) \,ds$. It is well known that the (AR)-condition is quite natural and important not only to ensure that an Euler–Lagrangian functional has the mountain pass geometry, but also to guarantee that the Palais–Smale sequences of the functional are bounded. However, this condition is very restrictive and eliminates many nonlinearities. Thus, many researchers have tried to drop the (AR)-condition for elliptic equations involving the *p*-Laplacian; see e.g. [21–24].

The purpose of this paper is to study the existence of weak solutions for the problem (P) without the (AR)-condition as observing various assumptions for the nonlinear term *f* compare to result in [19]. In particular, following Remark 1.8 in [21], there are many examples which do not fulfill the condition of *f* given in [22, 23, 25]. On the other hand, in the case of the whole space $\mathbb {R}^{N}$, the main difficulty of this problem is the lack of compactness for the Sobolev theorem and we introduce the potential *V* to the equation. To be precise, we prove the existence of weak solutions for the quasilinear Choquard equation (P) under the Cerami condition, as a weak version of the Palais–Smale condition. To do this, first, we use the uniform boundedness of the convolution part, $\vert I_{\alpha}*F \vert < \infty$ for our analysis (see Section 3.1 for a detailed description) and thus the property of $(S_{+})$ type operator with this uniform estimate gives a lot of help when we choose Cerami sequences. Second, we show the multiplicity of weak solutions to the quasilinear Choquard equation (P) via the fountain theorem to obtain the infinitely many weak solutions. Third, we establish the existence of a sequence of weak solutions for the problem (P) converging to zero to obtain the $L^{\infty}$-bound of weak solutions to the problem (P) based on an iteration method. To the best of our knowledge, there were no such existence results for our problem in this situation.

## Preliminaries

Let $1< p< N$ and $p^{*}:=Np/(N-p)$ denote the Sobolev conjugate of *p*. Suppose that

(V) $V\in C(\mathbb {R}^{N},\mathbb {R})$, $\inf_{x\in\mathbb {R}^{N}}V(x):=V_{0}>0$, $\operatorname{meas} \{x\in\mathbb {R}^{N}:V(x)\le M \} <+\infty$ for all $M\in\mathbb {R}$.

Define the linear subspace

$$X:= \biggl\{ u\in W^{1,p}\bigl({\mathbb {R}}^{N}\bigr):{ \int_{\mathbb {R}^{N}} { \vert \nabla u \vert ^{p} \,dx + \int_{\mathbb {R}^{N}}{V(x) \vert u \vert ^{p}}} \,dx}< \infty \biggr\} . $$

Then *X* is a reflexive separable Banach space with the norm

$$\Vert u \Vert _{X}= \biggl( \int_{\mathbb {R}^{N}} { \vert \nabla u \vert ^{p}} \,dx + \int_{\mathbb {R}^{N}} {V(x) \vert u \vert ^{p}} \,dx \biggr)^{\frac{1}{p}}, $$

which is equivalent to the norm $\Vert \cdot \Vert _{W^{1,p}({\mathbb {R}}^{N})}$ given by

$$\Vert u \Vert _{W^{1,p}({\mathbb {R}}^{N})}= \biggl( \int_{\mathbb {R}^{N}} { \vert \nabla u \vert ^{p}} \,dx + \int_{\mathbb {R}^{N}} { \vert u \vert ^{p}} \,dx \biggr)^{\frac{1}{p}}. $$

We recall the well-known embedding results in [21, Lemma 2.1]; see also [26].

### Lemma 2.1

*The following statements hold*:

(i) *There is a continuous embedding* $W^{1,p}({\mathbb {R}}^{N})\hookrightarrow L^{s}({\mathbb {R}}^{N})$ *for any* $s \in [p, p^{*}]$.
(ii) *If* *V* *satisfies the assumption* (V), *then there is a compact embedding* $X\hookrightarrow L^{s}({\mathbb {R}}^{N})$ *for any* $s \in[p, p^{*})$.

Throughout this paper, let *X* be the completion of $C_{0}^{\infty}(\mathbb {R}^{N}, \mathbb {R})$, and $X^{*}$ be a dual space of *X*. Furthermore, $\langle\cdot,\cdot \rangle$ denotes the pairing of *X* and its dual $X^{*}$. All generic constants will be denoted by *C*, which may vary from line to line.

### Definition 2.2

We say that $u\in X$ is a weak solution of the problem (P) if

2.1 $$ \int_{\mathbb {R}^{N}} \vert \nabla u \vert ^{p-2}\nabla u \cdot\nabla v \,dx + \int_{\mathbb {R}^{N}} V(x)|u|^{p-2}u v \,dx=\lambda \int_{\mathbb {R}^{N}}\bigl(I_{\alpha}*F(u)\bigr)f(u) v \,dx $$

for all $v \in X$.

Let us define the functional $\Phi: X \to\mathbb {R}$ by

$$\Phi(u)=\frac{1}{p} \int_{\mathbb {R}^{N}} \vert \nabla u \vert ^{p} \,dx+ \frac {1}{p} \int_{\mathbb {R}^{N}} V(x)|u|^{p} \,dx. $$

Under the assumption (V), it is obvious that the functional Φ is well defined on *X*, $\Phi\in C^{1}(X,\mathbb {R})$ and its Fréchet derivative is given by

$$ \bigl\langle {\Phi^{\prime}(u),v}\bigr\rangle = \int_{\mathbb {R}^{N}} \vert \nabla u \vert ^{p-2}\nabla u \cdot\nabla v \,dx + \int_{\mathbb {R}^{N}} V(x)|u|^{p-2}u v \,dx. $$

We suppose that the following assumptions hold:

1. $F\in C^{1}({\mathbb {R}},{\mathbb {R}})$.
2. There exist a constant $\sigma>0$ and $1< p< q_{1}\le q_{2}< \frac{\alpha p}{N-p}$ such that for all $t \in\mathbb {R}$,
  $$\bigl\vert f(t) \bigr\vert \le\sigma\bigl( \vert t \vert ^{q_{1}-1}+ \vert t \vert ^{q_{2}-1}\bigr). $$
3. There exists $\delta>0$ such that
  $$F(t)\leq0\quad \text{and}\quad \vert t \vert < \delta. $$
4. $\lim_{ \vert t \vert \to\infty}{\frac {F(t)}{ \vert t \vert ^{ p}}}=\infty$.
5. There exist $c_{0}\ge0$, $r_{0}\ge0$, and $\kappa> \frac{N}{p}$ such that
  $$\bigl\vert F(t) \bigr\vert ^{\kappa}\le c_{0} \vert t \vert ^{\kappa p}{\mathfrak {F}}(t) $$
  for all $t\in\mathbb {R}$ and $\vert t \vert \ge r_{0}$, where ${\mathfrak {F}}(t):=\frac{1}{p}f(t)t-\frac{1}{2}F(t)\ge0$.
6. $f(-t)=-f(t)$ holds for all $t\in\mathbb {R}$.

To comment on the assumptions about the nonlinearity *f*, we would like to recall an important inequality due to [1].

Let $s, r > 1$ and $\alpha< p $ with $1/s + (N-\alpha)/N + 1/r = 2$. Let $g\in L^{s} (\mathbb {R}^{N})$ and $h\in L^{r}(\mathbb {R}^{N})$. Then there exists a sharp constant $C(s, N, \alpha, r)$, independent of *g* and *h*, such that

$$\int_{\mathbb {R}^{N}} \int_{\mathbb {R}^{N}} \frac{g(x)h(y)}{ \vert x-y \vert ^{N-\alpha }} \,dx\,dy \leq C(s, N, \alpha, r) \Vert g \Vert _{L^{s}(\mathbb {R}^{N})} \Vert h \Vert _{L^{r}(\mathbb {R}^{N})}. $$

In particular, $F(t) = |t|^{q_{1}}$ for some $q_{1} > 0$. By the Hardy–Littlewood–Sobolev inequality,

$$\int_{\mathbb {R}^{N}} \int_{\mathbb {R}^{N}} \frac{F(u(x))F(u(y))}{ \vert x-y \vert ^{N-\alpha}} \,dy \,dx $$

is well defined if $F\in L^{s} (\mathbb {R}^{N} )$ for $s > 1$ is defined by

$$\frac{2}{s} + \frac{N-\alpha}{N} = 2. $$

Since $u \in W^{1,p}(\mathbb {R}^{N} )$, we must require that $sq_{1}\in[p, p^{*}]$. For the subcritical case, we must assume

$$\frac{p}{2} \biggl(2 -\frac{N-\alpha}{N} \biggr) < q_{1} \leq q_{2} < \frac {p^{*}}{2} \biggl(2- \frac{N-\alpha}{N} \biggr). $$

Next we define the functional $\Psi:X\to\mathbb {R}$ by

$$\Psi(u)=\frac{1}{2} \int_{\mathbb {R}^{N}}\bigl(I_{\alpha}*F(u)\bigr)F(u) \,dx. $$

Then it is easy to check that $\Psi\in C^{1}(X,\mathbb {R})$ and its Fréchet derivative is

$$ \bigl\langle \Psi^{\prime}(u),v \bigr\rangle = \int_{\mathbb {R}^{N}}\bigl(I_{\alpha}*F(u)\bigr)f(u)v \,dx $$

for any $u,v \in X$. Also we define the functional $\mathcal{I}_{\lambda}:X\to\mathbb {R}$ by

$$\mathcal{I}_{\lambda}(u)=\Phi(u)-\lambda\Psi(u). $$

Then it follows that the functional $\mathcal{I}_{\lambda}\in C^{1}(X,\mathbb {R})$ and its Fréchet derivative is

$$\bigl\langle \mathcal{I}_{\lambda}^{\prime}(u),v \bigr\rangle = \int_{\mathbb {R}^{N}} \vert \nabla u \vert ^{p-2}\nabla u \cdot\nabla v \,dx + \int _{\mathbb {R}^{N}} V(x)|u|^{p-2}u v \,dx-\lambda \int_{\mathbb {R}^{N}}\bigl(I_{\alpha}*F(u)\bigr)f(u) v \,dx $$

for any $u,v\in X$.

According to similar arguments in [27, Theorem 4.1], the following lemma is easily checked, and thus we omit the proof. That is, the operator $\Phi^{\prime}$ is of type $(S_{+})$; see [28].

### Lemma 2.3

*Assume that the assumption* (V) *holds*. *Then the functional* $\Phi:X\to\mathbb {R}$ *is convex and weakly lower semicontinuous on* *X*. *Moreover*, *the operator* $\Phi^{\prime}$ *is of type* $(S_{+})$, *i*.*e*., *if* $u_{n}\rightharpoonup u$ *in* *X* *and* $\lim\sup_{n\to \infty} \langle\Phi^{\prime}(u_{n})-\Phi^{\prime}(u), u_{n}-u \rangle\le0$, *then* $u_{n}\to u$ *in* *X* *as* $n\to\infty$.

In our setting, we need the following lemma according to a similar argument in [29, Lemma 3.2]. We give a detailed proof for the convenience of the reader.

### Lemma 2.4

*Assume that* (V) *and* (F1)*–*(F2) *hold*. *Then* Ψ *and* $\Psi^{\prime}$ *are weakly strongly continuous on* *X*.

### Proof

See the Appendix. □

## Existence of weak solutions

In this section, we shall give the proof of the existence of nontrivial weak solutions for the problem (P), by applying the mountain pass theorem and the fountain theorem.

With the aid of Lemmas 2.3 and 2.4, we prove that the energy functional $\mathcal{I}_{\lambda}$ satisfies the Cerami condition ($(C)_{c}$-condition for short), i.e., for $c\in\mathbb {R}$, any sequence $\{u_{n} \}\subset X$ such that

$$\mathcal{I}_{\lambda}(u_{n})\to c \quad \text{and}\quad \bigl\Vert \mathcal{I}_{\lambda}^{\prime}(u_{n}) \bigr\Vert _{X^{*}}\bigl(1+ \Vert u_{n} \Vert _{X}\bigr) \to 0 \quad \text{as } n\to\infty $$

has a convergent subsequence. This plays a key role in obtaining the existence of a nontrivial weak solution for the given problem.

Before investigating a crucial lemma, we note that following [19], there exists $\mathcal{M}>0$, such that

3.1 $$ \bigl\vert I_{\alpha}*F(v) \bigr\vert \leq\mathcal{M} \quad \mbox{for } v\in X. $$

Indeed, by the assumption (F2),

$$\begin{aligned} \bigl\vert I_{\alpha}*F(v) \bigr\vert &= \biggl\vert \int_{\mathbb {R}^{N}} \frac{F(v)}{ \vert x-y \vert ^{N-\alpha}} \,dy \biggr\vert \\ &= \biggl\vert \int_{ \vert x-y \vert \leq1} \frac {F(v)}{ \vert x-y \vert ^{N-\alpha}} \,dy \biggr\vert + \biggl\vert \int_{ \vert x-y \vert \geq1} \frac {F(v)}{ \vert x-y \vert ^{N-\alpha}} \,dy \biggr\vert \\ &\leq\sigma \int_{ \vert x-y \vert \leq1} \frac{{ \vert v \vert }^{q_{1}}+{ \vert v \vert }^{q_{2}}}{ \vert x-y \vert ^{N-\alpha}} \,dy + \sigma \int_{ \vert x-y \vert \geq1} \bigl({ \vert v \vert }^{q_{1}}+{ \vert v \vert }^{q_{2}}\bigr) \,dy \\ &\leq\sigma \int_{ \vert x-y \vert \leq1} \frac{{ \vert v \vert }^{q_{1}}+{ \vert v \vert }^{q_{2}}}{ \vert x-y \vert ^{N-\alpha}} \,dy + C, \end{aligned}$$

where we use the fact that $p< q_{1}\leq q_{2}< p^{*}$. Choosing $t_{1} \in(N/\alpha, Np/(N-p)q_{1})$ and $t_{2} \in(N/\alpha, Np/(N-p)q_{2})$, it follows from Hölder’s inequality,

$$\begin{aligned} \int_{ \vert x-y \vert \leq1} \frac{{ \vert v \vert }^{q_{1}}}{ \vert x-y \vert ^{N-\alpha}} \,dy &\leq { \biggl( \int_{ \vert x-y \vert \leq1} { \vert v \vert }^{t_{1} q_{1}} \,dy \biggr)}^{\frac{1}{t_{1}}}{ \biggl( \int_{ \vert x-y \vert \leq1} \frac{1}{ \vert x-y \vert ^{\frac{t_{1} (N-\alpha)}{t_{1}-1}}} \,dy \biggr)}^{\frac{t_{1}-1}{t_{1}}} \\ &\leq C{ \biggl( \int_{ \vert r \vert \leq1} \vert r \vert ^{N-1-\frac{t_{1}(N-\alpha)}{t_{1}-1}} \,dr \biggr)}^{\frac{t_{1}-1}{t_{1}}}. \end{aligned}$$

Similarly, we get

$$ \int_{ \vert x-y \vert \leq1} \frac{{ \vert v \vert }^{q_{2}}}{ \vert x-y \vert ^{N-\alpha}} \,dy \leq C{ \biggl( \int_{ \vert r \vert \leq1} \vert r \vert ^{N-1-\frac{t_{2}(N-\alpha)}{t_{2}-1}} \,dr \biggr)}^{\frac{t_{2}-1}{t_{2}}}. $$

Since both $N-1-\frac{t_{i}(N-\alpha)}{t_{i}-1} > -1$, for $i= 1,2$, there is a constant $C >0 $ such that

$$\int_{ \vert x-y \vert \leq1} \frac{{ \vert v \vert }^{q_{1}}+{ \vert v \vert }^{q_{2}}}{ \vert x-y \vert ^{N-\alpha}} \,dy \leq C \quad \mbox{for all } x \in\mathbb {R}^{N}. $$

Hence this inequality implies the uniform boundedness (3.1) for the convolution part.

### Existence of weak solutions: approach to the mountain pass theorem

We give the following result to show that the energy functional $\mathcal{I}_{\lambda}$ satisfies the geometric conditions of the mountain pass theorem based on the idea of Lemma 3.2 in [30].

#### Lemma 3.1

*Assume that* (V) *and* (F1)*–*(F4) *hold*. *Then the geometric conditions in the mountain pass theorem hold*, *i*.*e*.,

1. $u=0$ *is a strict local minimum for* $\mathcal{I}_{\lambda}(u)$,
2. $\mathcal{I}_{\lambda}(u)$ *is unbounded from below on* *X*.

#### Proof

In view of the assumption (F3), $u=0$ is a strict local minimum for $\mathcal{I}_{\lambda}(u)$. Next we show that the condition (2) holds. By the assumption (F4), we can take $s_{0}$ such that $F(s_{0})\neq0$ and we have to find

$$\int_{\mathbb {R}^{N}}\bigl({I}_{\alpha}*F(s_{0} \mathbf{1}_{B_{1}})\bigr)F(s_{0}\mathbf{ 1}_{B_{1}}) \,dx=F(s_{0})^{2} \int_{B_{1}} \int_{B_{1}}I_{\alpha}(x-y)\,dx\,dy>0, $$

where $B_{r}$ denotes the open ball centered at the origin with radius *r* and $\mathbf{1}_{A}$ denotes the standard indicator function of a set *A*.

Due to the density theorem, there will be $v_{0}\in X$ with

$$\int_{\mathbb {R}^{N}}\bigl({I}_{\alpha}*F(v_{0}) \bigr)F(v_{0}) \,dx>0. $$

Now, for $t>0$, we define a function $v_{t}:\mathbb {R}^{N}\to\mathbb {R}$ for $x\in\mathbb {R}^{N}$ by $v_{t}(x):=v_{0} (\frac{x}{t} )$. This function verifies

$$\mathcal{I}_{\lambda}(v_{t})=\frac{t^{N-p}}{p} \int_{\mathbb {R}^{N}} \vert \nabla v_{0} \vert ^{p} \,dx +\frac{t^{N}}{p} \int _{\mathbb {R}^{N}} V(x)|v_{0}|^{p} \,dx- \frac{\lambda t^{N+\alpha}}{2} \int_{\mathbb {R}^{N}}\bigl(I_{\alpha}*F(v_{0}) \bigr)F(v_{0}) \,dx $$

for sufficiently large *t*. Therefore, we assert that $\mathcal{I}_{\lambda}(v_{t})\to-\infty$ as $t \to\infty$. Hence we conclude that the functional $\mathcal{I}_{\lambda}$ is unbounded from below. This completes the proof. □

#### Lemma 3.2

*Assume that* (V), (F1)*–*(F2), *and* (F4)*–*(F5) *hold*. *Then the functional* $\mathcal{I}_{\lambda}$ *satisfies the* $(C)_{c}$-*condition for any* $\lambda>0$.

#### Proof

For $c\in\mathbb {R}$, let $\{u_{n}\}$ be a $(C)_{c}$-sequence in *X*, that is,

$$ \mathcal{I}_{\lambda}(u_{n})\to c \quad \text{and} \quad \bigl\Vert \mathcal{I}_{\lambda}^{\prime}(u_{n}) \bigr\Vert _{X^{*}}\bigl(1+ \Vert u_{n} \Vert _{X}\bigr)\to 0 \quad \text{as } n\to\infty. $$

This says that

3.2 $$ c = \mathcal{I}_{\lambda}(u_{n})+o(1)\quad \text{and}\quad \bigl\langle \mathcal{I}_{\lambda}^{\prime}(u_{n}), u_{n} \bigr\rangle =o(1), $$

where $o(1)\to0$ as $n\to\infty$. It follows from Lemmas 2.3 and 2.4 that $\mathcal{I}_{\lambda}^{\prime}$ is of type $(S_{+})$. Since $\mathcal{I}_{\lambda}^{\prime}$ is of type $(S_{+})$ and *X* is reflexive, it suffices to prove that the sequence $\{u_{n}\}$ is bounded in *X*. We argue by contradiction. Suppose that the sequence $\{u_{n} \}$ is unbounded in *X*. Then we may assume that $\Vert u_{n} \Vert _{X}>1$ and $\Vert u_{n} \Vert _{X}\to\infty$ as $n\to\infty$. Define a sequence $\{w_{n} \}$ by $w_{n}={u_{n}}/{ \Vert u_{n} \Vert _{X}}$. It is clear that $\{w_{n} \} \subset X$ and $\Vert w_{n} \Vert _{X}=1$. Hence, up to a subsequence, still denoted by $\{w_{n} \}$, we obtain $w_{n}\rightharpoonup w$ in *X* as $n\to\infty$ and note that

3.3 $$ w_{n}(x) \to w(x) \quad \text{a.e. in }\mathbb {R}^{N} \quad \text{and}\quad w_{n} \to w \quad \text{in }L^{s}\bigl(\mathbb {R}^{N}\bigr) \text{ as } n\to\infty $$

for $p\leq s< p^{*}$. According to (3.1), we obtain

3.4 $$\begin{aligned} c&=\mathcal{I}_{\lambda}(u_{n})+o(1) \\ &=\frac{1}{p} \int_{\mathbb {R}^{N}} \vert \nabla u_{n} \vert ^{p} \,dx +\frac {1}{p} \int_{\mathbb {R}^{N}} V(x)|u_{n}|^{p} \,dx- \frac{\lambda}{2} \int _{\mathbb {R}^{N}}\bigl(I_{\alpha}*F(u_{n}) \bigr)F(u_{n}) \,dx+o(1) \\ &=\frac{1}{p} \Vert u_{n} \Vert _{X}^{p}- \frac{\lambda}{2} \int_{\mathbb {R}^{N}} \bigl(I_{\alpha}*F(u_{n}) \bigr)F(u_{n}) \,dx+o(1). \end{aligned}$$

Since $\Vert u_{n} \Vert _{X}\to\infty$ as $n\to\infty$, we have

3.5 $$ \frac{1}{2} \int_{\mathbb {R}^{N}}{ \bigl(I_{\alpha}*F(u_{n}) \bigr)F(u_{n})} \,dx=\frac{1}{ p\lambda } \Vert u_{n} \Vert _{X}^{p}- \frac{c}{\lambda}+\frac {o(1)}{\lambda} \to \infty\quad \text{as } n\to\infty. $$

In addition, it follows from Eq. (3.2) that

$$ \frac{1}{p} \Vert u_{n} \Vert _{X}^{p}= \frac{\lambda}{2} \int_{\mathbb {R}^{N}}{\bigl(I_{\alpha}*F(u_{n}) \bigr)F(u_{n}) } \,dx+c-o(1) $$

for sufficiently large *n*. The assumption (F4) implies that there exists $t_{0}>1$ such that ${F(t)}>{ \vert t \vert ^{p}}$ for all $\vert t \vert >t_{0}$. From the assumptions (F1) and (F2), there exists $\mathcal{C}>0$ such that $\vert F(t) \vert \leq \mathcal{C}$ for all $t\in[-t_{0},t_{0}]$. Therefore we can choose a real number $\mathcal{C}_{0}$ such that $F(t)\geq\mathcal{C}_{0}$ for all $t\in\mathbb {R}$, and thus

$$ \frac{F(u_{n})-\mathcal{C}_{0}}{\frac{1}{p } \Vert u_{n} \Vert _{X}^{p}}\geq 0 $$

for all $n\in\mathbb {N}$. Therefore there exists a real number $\mathcal{C}_{1}$ such that

$$ \frac{F(u_{n})-\mathcal{C}_{0}}{\frac{1}{p } \Vert u_{n} \Vert _{X}^{p}}\geq \frac{(I_{\alpha}*F(u_{n}))F(u_{n})-\mathcal{C}_{1}}{\frac{1}{p } \Vert u_{n} \Vert _{X}^{p}}\geq0. $$

Set $\Omega_{1}= \{ x\in\mathbb {R}^{N} : w(x)\neq0 \}$. By the convergence (3.3), we know that

$$\bigl\vert u_{n}(x) \bigr\vert = \bigl\vert w_{n}(x) \bigr\vert \Vert u_{n} \Vert _{X}\to\infty \quad \text{as } n\to\infty $$

for all $x\in\Omega_{1}$. So then it follows from the assumption (F4) and Hölder’s inequality that, for all $x\in\Omega_{1}$, we have

3.6 $$ \lim_{n\to\infty}{\frac{(I_{\alpha}*F(u_{n}))F(u_{n})}{\frac{1}{p } \Vert u_{n} \Vert _{X}^{p}}}=\lim _{n\to\infty}{\frac {(I_{\alpha }*F(u_{n}))F(u_{n})}{\frac{1}{p } \vert u_{n}(x) \vert ^{p}}} \bigl\vert w_{n}(x) \bigr\vert ^{p}= \infty. $$

Hence we get $\operatorname{meas}(\Omega_{1})=0$. Indeed, if $\operatorname{meas}(\Omega_{1})\neq0$, according to (3.5)–(3.6) and Fatou’s lemma, we obtain

$$\begin{aligned} \frac{1}{\lambda} &={\liminf_{n\to\infty}{ \frac{ \frac {1}{2}{\int_{\mathbb {R}^{N}}{(I_{\alpha}*F(u_{n}))F(u_{n})} \,dx}}{\frac{\lambda}{2}\int _{\mathbb {R}^{N}}{(I_{\alpha}*F(u_{n}))F(u_{n})} \,dx+c-o(1)}}} \\ &={\liminf_{n\to\infty} \int_{\mathbb {R}^{N}}{\frac{(I_{\alpha}*F(u_{n}))F(u_{n})}{\frac{1}{p } \Vert u_{n} \Vert _{X}^{p}}}} \,dx \\ &\geq {\liminf_{n\to\infty} \int_{\Omega_{1}}{\frac{(I_{\alpha }*F(u_{n}))F(u_{n})}{\frac{1}{p } \Vert u_{n} \Vert _{X}^{p}}}} \,dx - \liminf _{n\to\infty}{ \int_{\Omega_{1}}{\frac{\mathcal {C}_{1}}{\frac{1}{p } \Vert u_{n} \Vert _{X}^{p}}} \,dx} \\ &\geq \liminf_{n\to\infty}{ \int_{\Omega_{1}}{\frac{(I_{\alpha }*F(u_{n}))F(u_{n})-\mathcal{C}_{1}}{\frac{1}{p } \Vert u_{n} \Vert _{X}^{p}}} \,dx} \\ &\ge \int_{\Omega_{1}}{\liminf_{n\to\infty}{ \frac{(I_{\alpha }*F(u_{n}))F(u_{n})-\mathcal{C}_{1}}{\frac{1}{p } \Vert u_{n} \Vert _{X}^{p}}}} \,dx \\ &\ge \int_{\Omega_{1}}{\liminf_{n\to\infty}{ \frac{(I_{\alpha }*F(u_{n}))F(u_{n})}{\frac{1}{p } \Vert u_{n} \Vert _{X}^{p}}}} \,dx -{ \int_{\Omega_{1}}\limsup_{n\to\infty}{\frac{\mathcal {C}_{1}}{\frac{1}{p } \Vert u_{n} \Vert _{X}^{p}}} \,dx} \\ &=\infty, \end{aligned}$$

from which we deduce a contradiction. Thus $w(x)=0$ for almost all $x\in\mathbb {R}^{N}$. Using (3.2), we get

3.7 $$\begin{aligned} c+1 \ge&\mathcal{I}_{\lambda}(u_{n})-\frac{1}{p} \bigl\langle \mathcal {I}_{\lambda}^{\prime}(u_{n}),u_{n} \bigr\rangle \\ =&\frac{1}{p} \int_{\mathbb {R}^{N}} \vert \nabla u_{n} \vert ^{p} \,dx +\frac {1}{p} \int_{\mathbb {R}^{N}} V(x)|u_{n}|^{p} \,dx- \frac{\lambda}{2} \int _{\mathbb {R}^{N}}\bigl(I_{\alpha}*F(u_{n}) \bigr)F(u_{n}) \,dx \\ &{} -\frac{1}{p} \int_{\mathbb {R}^{N}} \vert \nabla u_{n} \vert ^{p} \,dx -\frac{1}{p} \int_{\mathbb {R}^{N}} V(x)|u_{n}|^{p} \,dx+ \frac{\lambda}{p} \int_{\mathbb {R}^{N}}\bigl(I_{\alpha}*F(u_{n}) \bigr)f(u_{n}) u_{n} \,dx \\ =& \lambda \int_{\mathbb {R}^{N}}\bigl(I_{\alpha}*F(u_{n})\bigr){ \mathfrak {F}}(u_{n}) \,dx\quad \mbox{for }n\text{ large enough}. \end{aligned}$$

Let us define $\Omega_{n}(a,b):=\{ x\in\mathbb {R}^{N} : a \le \vert u_{n}(x) \vert < b \}$ for $a \geq0$. The convergence (3.3) means that

3.8 $$ w_{n}\to0\quad \text{in } L^{r}\bigl( \mathbb {R}^{N}\bigr)\quad \text{and}\quad w_{n}(x)\to0\quad \text{a.e. in } \mathbb {R}^{N} \text{ as } n\to\infty $$

for $p \leq r < p^{*}$. Hence by the relation (3.4) we get

3.9 $$ 0< \frac{2}{\lambda p} \le\limsup_{n\to\infty} \int_{\mathbb {R}^{N}}{\frac{ \vert I_{\alpha}*F(u_{n}) \vert \vert F(u_{n}) \vert }{ \Vert u_{n} \Vert _{X}^{p}}} \,dx. $$

On the other hand, from the assumption (F2) and Eq. (3.3), it follows that

3.10 $$\begin{aligned} & \int_{\Omega_{n}(0,r_{0})}{\frac{(I_{\alpha}*F(u_{n}))F(u_{n})}{ \Vert u_{n} \Vert _{X}^{p}}} \,dx \\ &\quad \le\mathcal{M} \int_{\Omega_{n}(0,r_{0})}{\frac{ \vert F(u_{n}) \vert }{ \Vert u_{n} \Vert _{X}^{p}}} \,dx \\ &\quad \le\mathcal{M}\sigma \int_{\Omega_{n}(0,r_{0})} \biggl(\frac{ \vert u_{n}(x) \vert ^{q_{1}}}{q_{1} \Vert u_{n} \Vert _{X}^{p}} + \frac{ \vert u_{n}(x) \vert ^{q_{2}}}{{q_{2}} \Vert u_{n} \Vert _{X}^{p}} \biggr) \,dx \\ &\quad \le\mathcal{M}\sigma \int_{\Omega_{n}(0,r_{0})} \biggl(\frac{ \vert u_{n}(x) \vert ^{q_{1}-p}}{q_{1}} \bigl\vert w_{n}(x) \bigr\vert ^{p} + \frac{ \vert u_{n}(x) \vert ^{q_{2}-p}}{q_{2}} \bigl\vert w_{n}(x) \bigr\vert ^{p} \biggr) \,dx \\ &\quad \le\mathcal{M}\sigma\bigl({r_{0}}^{q_{1}-p}+{r_{0}}^{q_{2}-p} \bigr) \int_{\mathbb {R}^{N}} { \bigl\vert w_{n}(x) \bigr\vert ^{p}} \,dx \to0\quad \mbox{as } n\to \infty \end{aligned}$$

due to $p< q_{1}\leq q_{2}$. Set $\kappa^{\prime}=\kappa/ (\kappa-1)$. Since $\kappa> N / p$, we get $p < \kappa^{\prime} p < p^{*}$. Hence, it follows from (F5), (3.7), and (3.8) that

3.11 $$\begin{aligned} & \int_{\Omega_{n}(r_{0},\infty)}{\frac{ \vert I_{\alpha }*F(u_{n}) \vert \vert F(u_{n}) \vert }{ \Vert u_{n} \Vert _{X}^{p}}} \,dx \\ &\quad = \int_{\Omega_{n}(r_{0},\infty)}{\frac{ \vert I_{\alpha }*F(u_{n}) \vert \vert F(u_{n}) \vert }{ \vert u_{n}(x) \vert ^{p}} \bigl\vert w_{n}(x) \bigr\vert ^{p}} \,dx \\ &\quad \le \biggl\{ \int_{\Omega_{n}(r_{0},\infty)} \biggl(\frac{ \vert I_{\alpha}*F(u_{n}) \vert \vert F(u_{n}) \vert }{ \vert u_{n}(x) \vert ^{p}} \biggr)^{\kappa} \,dx \biggr\} ^{\frac{1}{\kappa}} \biggl\{ \int_{\Omega(r_{0},\infty)} \bigl\vert w_{n}(x) \bigr\vert ^{\kappa^{\prime} p} \,dx \biggr\} ^{\frac{1}{\kappa^{\prime}}} \\ &\quad \le c_{0}^{\frac{1}{\kappa}} \biggl\{ \int_{\Omega_{n}(r_{0},\infty )}{ \bigl\vert I_{\alpha}*F(u_{n}) \bigr\vert ^{\kappa}\mathfrak {F}}(u_{n}) \,dx \biggr\} ^{\frac{1}{\kappa}} \biggl\{ \int_{\mathbb {R}^{N}} \bigl\vert w_{n}(x) \bigr\vert ^{\kappa^{\prime}p} \,dx \biggr\} ^{\frac{1}{\kappa ^{\prime}}} \\ &\quad \le c_{0}^{\frac{1}{\kappa}}{\mathcal{M}}^{\frac{\kappa-1}{\kappa }} \biggl\{ \int_{\Omega_{n}(r_{0},\infty)}{ \bigl\vert I_{\alpha }*F(u_{n}) \bigr\vert \mathfrak {F}}(u_{n}) \,dx \biggr\} ^{\frac{1}{\kappa}} \biggl\{ \int_{\mathbb {R}^{N}} \bigl\vert w_{n}(x) \bigr\vert ^{\kappa^{\prime}p} \,dx \biggr\} ^{\frac{1}{\kappa ^{\prime}}} \\ &\quad \le c_{0}^{\frac{1}{\kappa}}{\mathcal {M}}^{\frac{\kappa-1}{\kappa}} \biggl( \frac{c+1}{\lambda} \biggr)^{\frac {1}{\kappa}} \biggl\{ \int_{\mathbb {R}^{N}} \bigl\vert w_{n}(x) \bigr\vert ^{\kappa^{\prime}p} \,dx \biggr\} ^{\frac{1}{\kappa ^{\prime}}} \to0 \quad \mbox{as } n\to \infty. \end{aligned}$$

Combining the estimates (3.10) with (3.11), we have

$$\begin{aligned} &\int_{\mathbb {R}^{N}}{\frac{ \vert I_{\alpha}*F(u_{n}) \vert \vert F(u_{n}) \vert }{ \Vert u_{n} \Vert _{X}^{p}}} \,dx \\ &\quad = \int_{\Omega_{n}(0,r_{0})}{\frac{ \vert I_{\alpha }*F(u_{n}) \vert \vert F(u_{n}) \vert }{ \Vert u_{n} \Vert _{X}^{p}}} \,dx+ \int_{\Omega_{n}(r_{0},\infty)}{\frac{ \vert I_{\alpha }*F(u_{n}) \vert \vert F(u_{n}) \vert }{ \Vert u_{n} \Vert _{X}^{p}}} \,dx \\ &\quad \to0 \quad \text{as } n\to\infty, \end{aligned}$$

which contradicts (3.9). This completes the proof. □

Using Lemma 3.2, we prove the existence of a nontrivial weak solution for our problem under the assumptions.

#### Theorem 3.3

*Assume that* (V) *and* (F1)*–*(F5) *hold*. *Then the problem* (P) *has a nontrivial weak solution for all* $\lambda>0$.

#### Proof

Note that $\mathcal{I}_{\lambda}(0)=0$. In view of Lemma 3.1, the geometric conditions in the mountain pass theorem are fulfilled. And also, $\mathcal{I}_{\lambda}$ satisfies the $(C)_{c}$-condition for any $\lambda>0$ by Lemma 3.2. Hence, the problem (P) has a nontrivial weak solution for all $\lambda>0$. This completes the proof. □

### Existence of a sequence of weak solutions: approach to the fountain theorem

In this subsection, applying the fountain theorem in [31, Theorem 3.6] with the oddity on *f*, we investigate infinitely many weak solutions for the problem (P). For this purpose, let *W* be a reflexive and separable Banach space. Then there are $\{e_{n}\}\subseteq W$ and $\{f_{n}^{*}\}\subseteq W^{*}$ such that

$$W=\overline{\operatorname{span}\{e_{n}:n=1,2,\ldots\}},\qquad W^{*}=\overline {\operatorname{span}\bigl\{ f_{n}^{*}:n=1,2, \ldots\bigr\} }, $$

and

$$ \bigl\langle f^{*}_{i},e_{j} \bigr\rangle = \textstyle\begin{cases} 1 &\text{if } i=j, \\ 0 &\text{if } i\ne j. \end{cases} $$

Let us denote $W_{n}=\operatorname{span}\{e_{n}\}$, $Y_{k}=\bigoplus_{n=1}^{k}W_{n}$, and $Z_{k}= \overline{\bigoplus_{n=k}^{\infty}W_{n}}$.

#### Lemma 3.4

*Let* *X* *be a real reflexive Banach space*. *Suppose that* $I \in C^{1}(X,\mathbb {R})$ *satisfies the* ${(C)_{c}}$-*condition for any* $c>0$ *and* *I* *is even*. *If for each sufficiently large* $k \in\mathbb {N}$, *there exist* $\rho_{k}> \delta_{k}>0$ *such that the following conditions hold*:

1. $b_{k}:=\inf\{ I(u):u\in Z_{k}, \Vert u \Vert _{X}= \delta _{k}\}\to\infty$ *as* $k\to\infty$;
2. $a_{k}:=\max\{ I(u):u\in Y_{k}, \Vert u \Vert _{X}=\rho_{k}\} \le0$.

*Then the functional* *I* *has an unbounded sequence of critical values*, *i*.*e*., *there exists a sequence* $\{u_{n}\}\subset X$ *such that* $I^{\prime}(u_{n})=0$ *and* $I(u_{n})\to\infty$ *as* $n\to\infty$.

#### Theorem 3.5

*Assume that* (V), (F1)*–*(F2), *and* (F4)*–*(F6) *hold*. *Then*, *for any* $\lambda>0$, *the problem* (P) *possesses an unbounded sequence of nontrivial weak solutions* $\{u_{n}\}$ *in* *X* *such that* $\mathcal{I}_{\lambda}(u_{n})\to\infty$ *as* $n\to\infty$.

#### Proof

It is obvious that $\mathcal{I}_{\lambda}$ is an even functional and satisfies the $(C)_{c}$-condition. It suffices to show that there exist $\rho_{k}> \delta_{k}>0$ such that

1. $b_{k}:=\inf\{\mathcal{I}_{\lambda}(u):u\in Z_{k}, \Vert u \Vert _{X}= \delta_{k}\}\to\infty$ as $k\to\infty$;
2. $a_{k}:=\max\{\mathcal{I}_{\lambda}(u):u\in Y_{k}, \Vert u \Vert _{X}=\rho_{k}\}\le 0$ for *k* large enough.

Denote

$$\alpha_{k}:=\sup_{u \in Z_{k}, \Vert u \Vert _{X}=1} \bigl( \Vert u \Vert _{L^{q_{1}}(\mathbb {R}^{N})}+ \Vert u \Vert _{L^{q_{2}}(\mathbb{R}^{N})} \bigr). $$

Then we have $\alpha_{k} \to0$ as $k \to\infty$. In fact, assume to the contrary that there exist $\varepsilon_{0}>0$, $k_{0}\in\mathbb {N}$, and a sequence $\{u_{k}\}$ in $Z_{k}$ such that

$$\Vert u_{k} \Vert _{X}=1\quad \text{and}\quad \Vert u_{k} \Vert _{L^{q_{1}}(\mathbb{R}^{N})}+ \Vert u_{k} \Vert _{L^{q_{2}}(\mathbb{R}^{N})} \ge \varepsilon_{0} $$

for all $k \ge k_{0}$. By the boundedness of the sequence $\{u_{k}\}$ in *X*, we can find an element $u \in X$ such that $u_{k} \rightharpoonup u$ in *X* as $k\to\infty$ and

$$\bigl\langle {f_{j}^{*},u}\bigr\rangle =\lim _{k \to \infty}{\bigl\langle {f_{j}^{*},u_{k}} \bigr\rangle }=0 $$

for $j=1,2,\ldots $ . Thus we deduce $u=0$. However, we see that

$$\varepsilon_{0} \le\lim_{k \to \infty}{\bigl( \Vert u_{k} \Vert _{L^{q_{1}}(\mathbb{R}^{N})}+ \Vert u_{k} \Vert _{L^{q_{2}}(\mathbb {R}^{N})}\bigr)}= \Vert u \Vert _{L^{q_{1}}(\mathbb{R}^{N})}+ \Vert u \Vert _{L^{q_{2}}(\mathbb{R}^{N})}=0, $$

which is a contradiction.

For any $u \in Z_{k}$, we may suppose that $\Vert u \Vert _{X}>1$. According to the assumption (F2), we obtain

$$\begin{aligned} \mathcal{I}_{\lambda}(u) &=\frac{1}{p} \int_{\mathbb {R}^{N}} \vert \nabla u \vert ^{p} \,dx+ \frac{1}{p} \int_{\mathbb {R}^{N}}V(x) \vert u \vert ^{p} \,dx- \frac{\lambda}{2} \int_{\mathbb {R}^{N}}{\bigl(I_{\alpha }*F(u)\bigr)F(u) } \,dx \\ &\ge\frac{1}{p} \Vert u \Vert _{X}^{p}- \frac{\lambda}{2} \int_{\mathbb {R}^{N}}{ \bigl\vert I_{\alpha}*F(u) \bigr\vert \bigl\vert F(u) \bigr\vert } \,dx \\ &\ge\frac{1}{p} \Vert u \Vert _{X}^{p}- \frac{\lambda\mathcal {M}\sigma }{2} \int_{\mathbb {R}^{N}} \biggl(\frac{ \vert u \vert ^{q_{1}}}{q_{1}}+\frac{ \vert u \vert ^{q_{2}}}{q_{2}} \biggr) \,dx \\ &\ge\frac{1}{p} \Vert u \Vert _{X}^{p}- \frac{\lambda\mathcal {M}\sigma }{2q_{1}} \bigl( \Vert u \Vert _{L^{q_{1}}(\mathbb{R}^{N})}+ \Vert u \Vert _{L^{{q_{2}}}(\mathbb{R}^{N})} \bigr)^{q_{i}} \\ &\ge\frac{1}{p} \Vert u \Vert ^{p}_{X}- \frac{\lambda \mathcal{M}\sigma}{2q_{1}} \alpha_{k}^{q_{i}} \Vert u \Vert ^{q_{i}}_{X}, \end{aligned}$$

where $q_{i}$ is either $q_{1}$ or $q_{2}$. If we take

$$\delta_{k}= \biggl(\frac{{\lambda}\mathcal{M}\sigma q_{i}\alpha _{k}^{q_{i}}}{2q_{1}} \biggr)^{{1}/ (p-q_{i} )}, $$

then $\delta_{k}\to\infty$ as $k\to\infty$ because $p< q_{i}$ and $\alpha_{k}\to0$ as $k\to\infty$. Hence, if $u \in Z_{k}$ and $\Vert u \Vert _{X}=\delta_{k}$, then we conclude that

$$\mathcal{I}_{\lambda}(u)\ge \biggl(\frac{1}{ p}-\frac{1}{q_{i}} \biggr)\delta_{k}^{p}\to\infty\quad \text{as } k\to \infty. $$

This implies that the condition (1) holds.

The proof of the condition (2) proceeds analogously as in the proof of Theorem 1.3 of [25]. For the reader’s convenience, we give the proof. Assume that the condition (2) is not true. Then, for some *k* there exists a sequence $\{u_{n}\}$ in $Y_{k}$ such that

3.12 $$ \Vert u_{n} \Vert _{X}\to\infty\quad \text{as } n\to\infty\quad \text{and} \quad \mathcal{I}_{\lambda}(u_{n}) \ge0. $$

Set $w_{n}=u_{n}/ \Vert u_{n} \Vert _{X}$. Note that $\Vert w_{n} \Vert _{X}=1$. Since $\dim{Y_{k}}<\infty$, there exists $w\in Y_{k}\setminus\{0\}$ such that up to a subsequence,

$$\Vert w_{n}-w \Vert _{X}\to0 \quad \text{and}\quad w_{n}(x)\to w(x) $$

for almost all $x\in\mathbb {R}^{N}$ as $n\to\infty$. If $w(x)\neq 0$, then $\vert u_{n}(x) \vert \to\infty$ for all $x\in\mathbb {R}^{N}$ as $n\to\infty$. Hence we obtain by the assumption (F4) that

$$\lim_{n\to\infty}{\frac{F(u_{n})}{ \Vert u_{n} \Vert _{X}^{ p}}} = \lim_{n\to\infty}{ \frac{F(u_{n})}{ \vert u_{n} \vert ^{ p}} \bigl\vert w_{n}(x) \bigr\vert ^{p} } = \infty $$

for all $x\in\Omega_{2}:= \{ x\in\mathbb {R}^{N} : w(x)\neq0 \}$. A similar argument to (3.6) proves that

$$\int_{\Omega_{2}}{\frac{(I_{\alpha}*F(u_{n}))F(u_{n})}{ \Vert u_{n} \Vert _{X}^{p}}} \,dx\to \infty\quad \text{as }n \to\infty. $$

Therefore, we conclude that

$$\begin{aligned} \mathcal{I}_{\lambda}(u_{n})&= \frac{1}{p} \Vert u_{n} \Vert _{X}^{p}-\frac{\lambda}{2} \int_{\mathbb {R}^{N}}{\bigl(I_{\alpha}*F(u_{n}) \bigr)F(u_{n})} \,dx \\ &= \Vert u_{n} \Vert _{X}^{p} \biggl( \frac{1}{p}-\frac{\lambda}{2} \int _{\Omega _{2}}{\frac{(I_{\alpha}*F(u_{n}))F(u_{n})}{ \Vert u_{n} \Vert _{X}^{p}}} \,dx \biggr)\to -\infty\quad \text{as }n\to\infty, \end{aligned}$$

which contradicts (3.12). This completes the proof. □

### Existence of a sequence of weak solutions converging to zero

Now, we deal with the existence of a sequence of weak solutions converging to zero for the problem (P). First of all, we need the following additional assumptions for *f*:

(F7) $pF(t) - f(t)t > 0$ for $t\neq0$.
(F8) $\lim_{ \vert t \vert \to0}{\frac {f(t)}{ \vert t \vert ^{p-2}t}}=+\infty$.

From the assumptions above, we show the existence of a sequence of solutions for the problem (P) converging to zero in the $L^{\infty}$-norm based on the iteration method in [32, Theorem 4.1]. Since the problem (P) contains the potential term *V*, more sophisticated analysis has to be carefully carried out in comparison to the result in [32] (compare to [33] for the bounded domain).

#### Proposition 3.6

*Assume that* (V) *and* (F1)*–*(F2) *hold*. *If* *u* *is a weak solution of the problem* (P), *then* $u\in L^{\infty}({\mathbb {R}}^{N})$.

#### Proof

The proof is given in the Appendix. □

The following lemma is quoted from [34].

#### Lemma 3.7

*Let* $I\in C^{1}(X,{\mathbb {R}})$ *where* *X* *is a Banach space*. *Assume* *I* *satisfies the* $(\mathit{PS})$-*condition*, *is even and bounded from below*, *and* $I(0)=0$. *If for any* $n\in{\mathbb {N}}$, *there exist an* *n*-*dimensional subspace* $X_{n}$ *and* $\rho_{n}>0$ *such that*

$$\sup_{X_{n}\cap S_{\rho_{n}}}{I}< 0, $$

*where* $S_{\rho}:= \{ u \in X : \Vert u \Vert _{X}=\rho \}$, *then* *I* *has a sequence of critical values* $c_{n}<0$ *satisfying* $c_{n}\to 0$ *as* $n \to\infty$.

The following lemmas are quoted from [28, 35].

#### Lemma 3.8

*Let* $I\in C^{1}(X,{\mathbb {R}})$ *where* *X* *is a Banach space*. *Assume that* (F1)*–*(F2) *and* (F7) *hold*. *Then*

$$I(u)=0= \bigl\langle I^{\prime}(u),u \bigr\rangle \quad \textit{if and only if}\quad u=0. $$

#### Lemma 3.9

*Assume that* (F1)*–*(F2) *and* (F7)*–*(F8) *hold*. *Then there exist* $t_{0}>0$ *and* $\tilde{f}\in C^{1}(\mathbb {R},{\mathbb {R}})$ *such that* $\tilde{f}(t)$ *is odd in* *t* *and satisfy*

$$\begin{aligned}& \tilde{\mathcal {F}}(t):=p\tilde{F}(t)-\tilde{f}(t)t \ge0, \\& \tilde{\mathcal {F}}(t)=0\quad \textit{if and only if} \quad t\equiv0 \quad \textit{or}\quad |t| \ge2t_{0}, \end{aligned}$$

*where* $\frac{\partial}{\partial t}\tilde{F}(t)=\tilde{f}(t)$.

#### Proof

Let us define a cut-off function $\kappa\in C^{1}({\mathbb {R}}, {\mathbb {R}})$ satisfying $\kappa(t)=1$ for $|t|\le t_{0}$, $\kappa(t)=0$ for $|t| \ge2t_{0}$, $|\kappa^{\prime}(t)|\le2/t_{0}$, and $\kappa^{\prime}(t)t\le0$. So, we define

3.13 $$ {\tilde{F}}(t)=\kappa(t)F(t)+\bigl(1-\kappa(t)\bigr) \xi|t|^{p} \quad \text{and}\quad {\tilde{f}}(t)=\frac{\partial}{\partial t}{ \tilde{F}}(t), $$

where $\xi> 0$ is a constant. It is straightforward that

$$p{\tilde{F}}(t) - {\tilde{f}}(t)t = \kappa(t){\mathcal {F}}(t) - \kappa^{\prime}(t)tF(t) + \kappa^{\prime}(t)t\xi|t|^{p}, $$

where ${\mathcal {F}}(t):=pF(t)-f(t)t$. For $|t| \le t_{0}$ and $|t| \ge2t_{0}$ the conclusion follows. Due to (F8), we choose a sufficiently small $t_{0}>0$ such that $F(t)\ge\xi t^{p}$ for $t_{0} \le|t| \le2t_{0}$. By the assumption (F7) with the definition of *κ*, we get the conclusion. □

Now, for convenience of the reader, we prove the following result using Proposition 3.6 and Lemmas 3.7 and 3.9 (see e.g. [35, pp. 18–21]).

#### Theorem 3.10

*Assume that* (V), (F1)*–*(F2), *and* (F4)*–*(F8) *hold*. *Then there exists a positive constant* $\lambda^{*}$ *such that*, *for every* $\lambda\in[0,\lambda^{*})$, *the problem* (P) *has a sequence of weak solutions* $u_{n}$ *such that* $\Vert u_{n} \Vert _{L^{\infty }(\mathbb {R}^{N})}\to0$ *as* $n\to\infty$.

#### Proof

First of all, we claim that $\mathcal{I}_{\lambda}$ is coercive on *X*. Let $u\in X$ and $\Vert u \Vert _{X}>1$. For the given function *f*, we can modify and extend ${\tilde{f}}\in C^{1}(\mathbb {R},{\mathbb {R}})$ satisfying all properties listed in Lemma 3.9 with *ξ* such that $\lambda pC\xi\le1 $. And also by Lemma 3.9, it is easy to show that ${\tilde{\mathcal{I}}_{\lambda}}\in C^{1}(X,{\mathbb {R}})$ and is even on *X*. Moreover, it follows from (F2) that, for $|u(x)|\leq2t_{0}$ for a sufficiently small $t_{0}$, there exists a positive constant $M_{1}$ such that $|F(u)|\le M_{1} |u|^{p}$.

Set $\Omega_{3}:= \{ x\in\mathbb {R}^{N} : \vert u(x) \vert \le t_{0} \}$, $\Omega_{4}:= \{ x\in\mathbb {R}^{N} : t_{0} \le|u(x)| \le 2t_{0} \}$, and $\Omega_{5}:= \{ x\in\mathbb {R}^{N} : 2t_{0} \le |u(x)| \}$, where $t_{0}$ is given in Lemma 3.9. From (F8), (3.13), and the conditions of *κ*, we have

$$\begin{aligned} {\tilde{\mathcal{I}}}_{\lambda}(u) =& \frac{1}{p} \int_{\mathbb {R}^{N}} \vert \nabla u \vert ^{p} \,dx + \frac{1}{p} \int_{\mathbb {R}^{N}} V(x)|u|^{p} \,dx-\frac{\lambda}{2} \int_{\mathbb {R}^{N}}\bigl(I_{\alpha}*{\tilde{F}}(u)\bigr){\tilde{F}}(u) \,dx \\ \ge&\frac{1}{p} \int_{\mathbb {R}^{N}}|\nabla u|^{p} \,dx + \frac{1}{p} \int_{\mathbb {R}^{N}}{V(x) \vert u \vert ^{p}} \,dx - \frac{\lambda}{2} \int_{\Omega_{3}}{\bigl(I_{\alpha}*{\tilde{F}}(u)\bigr){\tilde{F}}(u)} \,dx \\ &{} - \frac{\lambda}{2} \int_{\Omega_{4}}\bigl(I_{\alpha}*{\tilde{F}}(u)\bigr) \bigl\{ \kappa(u)F(u)+\bigl(1-\kappa(u)\bigr)\xi|u|^{p} \bigr\} \,dx \\ &{}- \frac{\lambda}{2} \int_{\Omega_{5}}{\bigl(I_{\alpha}*{\tilde{F}}(u)\bigr)\xi |u|^{p}} \,dx \\ \ge&\frac{1}{p} \Vert u \Vert ^{p}_{X}- \frac{\lambda\mathcal{M}}{2} \int_{\Omega_{3}}{ \bigl\vert F(u) \bigr\vert } \,dx - \frac{\lambda\mathcal{M}}{2} \int_{\Omega_{4}} \bigl\vert F(u) \bigr\vert \,dx \\ &{}- \frac {\lambda\mathcal{M}}{2} \int_{\Omega_{4}}\xi|u|^{p} \,dx - \frac {\lambda\mathcal{M}}{2} \int_{\Omega_{5}}{\xi|u|^{p}} \,dx \\ \ge&\frac{1}{p} \Vert u \Vert ^{p}_{X}- \frac{\lambda\mathcal{M}M_{1}}{2} \int_{\Omega_{3}}{|u|^{p}} \,dx - \frac {\lambda\mathcal{M}M_{1}}{2} \int_{\Omega_{4}}{|u|^{p}} \,dx \\ &{}-\frac {\lambda\mathcal{M}}{2} \int_{\Omega_{4}}\xi|u|^{p} \,dx - \frac {\lambda\mathcal{M}}{2} \int_{\Omega_{5}}{\xi|u|^{p}} \,dx \\ \ge&\frac{1}{p} \Vert u \Vert ^{p}_{X} - \frac{\lambda\mathcal{M} ({M_{1}}+\xi )}{2V_{0}} \Vert u \Vert _{X}^{p}. \end{aligned}$$

If we set

$$\lambda^{*}:=\frac{2V_{0}}{p\mathcal{M} ({M_{1}}+\xi )}, $$

then for every $\lambda\in[0,\lambda^{*})$ we have ${\tilde{\mathcal{I}}}_{\lambda}$ is coercive, that is, ${\tilde{\mathcal{I}}_{\lambda}}(u)\to\infty$ as $\Vert u \Vert _{X}\to\infty$. By a standard argument, ${\tilde{\mathcal{I}}_{\lambda}}$ satisfies the $(\mathit{PS})_{c}$-condition. In order to apply Lemma 3.7, we only need to find for any $n\in{\mathbb {N}}$, a subspace $X_{n}$ and $\rho_{n}>0$ such that $\sup_{X_{n}\cap S_{\rho_{n}}}{{\tilde{\mathcal{I}}_{\lambda}}}<0$. For any $n\in{\mathbb {N}}$ we find *n* independent smooth functions $\phi_{i}$ for $i=1,\ldots,n$, and define $X_{n}:= \operatorname{span} \{ \phi_{1},\ldots,\phi_{n} \}$. Due to Lemma 3.9, when $\Vert u \Vert _{X}<1$ we have

3.14 $$\begin{aligned} {\tilde{\mathcal{I}}_{\lambda}}(u)&= \frac{1}{p} \int_{\mathbb {R}^{N}} \vert \nabla u \vert ^{p} \,dx + \frac{1}{p} \int_{\mathbb {R}^{N}} V(x)|u|^{p} \,dx-\frac{\lambda}{2} \int_{\mathbb {R}^{N}}\bigl(I_{\alpha}*{\tilde{F}}(u)\bigr){\tilde{F}}(u) \,dx \\ &\le\frac{1}{p} \Vert u \Vert _{X}^{p} - \frac{\lambda C}{2} \int _{\mathbb {R}^{N}}{\bigl(I_{\alpha}*{F}(u)\bigr)F(u)} \,dx. \end{aligned}$$

It follows from the assumption (F8) that, for a sufficiently large $M_{2}>0$, there exists $\delta_{0}>0$ such that $\vert t \vert <\delta_{0}$ implies $F(t)\ge\frac {M_{2}}{p} \vert t \vert ^{p}$ and

$$\int_{\mathbb {R}^{N}}{\bigl(I_{\alpha}*{F}(t)\bigr)F(t)} \,dx \ge \frac{M_{2}}{p} \int_{\mathbb {R}^{N}}{\bigl(I_{\alpha}*{F}(t)\bigr) \vert t \vert ^{p}} \,dx. $$

Combining this and the fact that all norms on $X_{n}$ are equivalent, choosing a suitable constant *C* and sufficiently small $\rho_{n}>0$, we can obtain by (3.14) that

$$ \sup_{X_{n}\cap S_{\rho_{n}}}{{\tilde{\mathcal{I}}_{\lambda}}}< 0. $$

By Lemma 3.7, we get a sequence $c_{n}<0$ for ${\tilde{\mathcal{I}}_{\lambda}}$ satisfying $c_{n}\to0$ when *n* goes to ∞. Then, for any $u_{n}\in X$ satisfying ${{\tilde{\mathcal{I}}_{\lambda}}}(u_{n})=c_{n}$ and ${{\tilde{\mathcal{I}}_{\lambda}}}^{\prime}(u_{n})=0$, the sequence $\{u_{n} \}$ is a $(\mathit{PS})_{0}$-sequence of ${{\tilde{\mathcal{I}}_{\lambda}}}(u)$ and $\{u_{n} \}$ has a convergent subsequence. Lemmas 3.8 and 3.9 imply that **0** is the only critical point with zero energy and the subsequence of $\{u_{n} \}$ has to converge to **0**. An indirect argument shows the sequence $\{u_{n} \}$ has to converge to **0**. On the other hand, we have $u_{n} \in C(\mathbb {R}^{N},\mathbb {R})$ due to Proposition 3.6. Since $\Vert u_{n} \Vert _{L^{\infty}(\mathbb {R}^{N})}\to0$, by Lemma 3.9 again, we deduce $\Vert u_{n} \Vert _{C(\mathbb {R}^{N})}\le t_{0}$. Thus $\{u_{n} \}$ are weak solutions of the problem (P). The proof is complete. □

## Conclusion

In this paper, we obtain the existence of nontrivial weak solutions for a quasilinear Choquard equation on the whole space $\mathbb{R}^{N}$ without (AR)-condition based on the uniform boundedness of the convolution part in the Choquard term driven by the Riesz potential. Moreover, the existence of infinitely many weak solutions is obtained via the fountain theorem. Lastly, we prove that our problem has a sequence of solutions converging to zero in the $L^{\infty}$-norm based on the iteration method.

Our arguments also allow one to prove Theorems 3.3 and 3.5 for the $p(x)$-Laplacian equation

$$ -\Delta_{p(x)} u+V(x)|u|^{p(x)-2}u=\bigl(I_{\alpha}\ast F(u)\bigr)f(u) \quad \text{in } \mathbb {R}^{N}, $$

where $p:\mathbb {R}^{N}\rightarrow \mathbb {R}$ is Lipschitz continuous with $1< p_{-} \le p_{+}< N$,

$$p_{+}=\sup_{x\in\mathbb {R}^{N}}p(x)\quad \mbox{and}\quad p_{-}=\inf _{x\in\mathbb {R}^{N}}p(x), $$

the potential *V* satisfying the assumption (V). Define the linear subspace

$$X= \biggl\{ u\in W^{1,p(\cdot)}\bigl(\mathbb {R}^{N}\bigr) : \int_{\mathbb {R}^{N}} \bigl( \vert \nabla u \vert ^{p(x)}+V(x) \vert u \vert ^{p(x)} \bigr) \,dx < +\infty \biggr\} $$

with the norm

$$\Vert u \Vert _{X}=\inf \biggl\{ \lambda>0: \int_{\mathbb {R}^{N}} \biggl( \biggl\vert \frac{\nabla u}{\lambda} \biggr\vert ^{p(x)}+V(x) \biggl\vert \frac{u}{\lambda} \biggr\vert ^{p(x)} \biggr) \,dx\le 1 \biggr\} , $$

which is equivalent to the following norm:

$$ \Vert u \Vert _{W^{1,p(\cdot)}(\mathbb {R}^{N})}= \Vert \nabla u \Vert _{L^{p(\cdot)}(\mathbb {R}^{N})}+ \Vert u \Vert _{L^{p(\cdot )}( \mathbb {R}^{N})}. $$

Under this circumstance, we introduce the functional $\mathcal{J}_{\lambda}:X\to\mathbb {R}$ by

$$\mathcal{J}_{\lambda}(u)=\Phi(u)-\lambda\Psi(u). $$

Then it follows that the functional $\mathcal{J}_{\lambda}\in C^{1}(X,\mathbb {R})$ and its Fréchet derivative is

$$\begin{aligned} \bigl\langle \mathcal{J}_{\lambda}^{\prime}(u),v \bigr\rangle =& \int_{\mathbb {R}^{N}} \vert \nabla u \vert ^{p(x)-2}\nabla u \cdot\nabla v \,dx + \int_{\mathbb {R}^{N}} V(x)|u|^{p(x)-2}u v \,dx \\ &{}-\lambda \int_{\mathbb {R}^{N}}\bigl(I_{\alpha}*F(u)\bigr)f(u) v \,dx \end{aligned}$$

for any $u,v\in X$. In order to show the boundedness of the Cerami sequence, we use the boundedness of the convolution part (3.1). For some properties of the variable exponent Sobolev space, we refer to [25]. And hence we omit the details proof.

## Appendix

In this section, we give proofs of Lemma 2.4 and Proposition 3.6 for the reader’s convenience. In fact, these are well-known results in this area.

### A.1 Proof of Lemma 2.4

#### Proof

Let $\{u_{n}\}$ be a sequence in *X* such that $u_{n}\rightharpoonup u$ in *X* as $n\to\infty$. Then $\{u_{n}\}$ is bounded in *X* and we know the embedding $X\hookrightarrow L^{s}(\mathbb {R}^{N})$ is compact for $p< s< p^{*}$. So we see that

$$ u_{n} \to u\quad \text{in } L^{q_{1}}\bigl(\mathbb {R}^{N}\bigr)\quad \text{and}\quad u_{n}\to u\quad \text{in } L^{q_{2}}\bigl(\mathbb {R}^{N}\bigr) \text{ as } n\to\infty. $$

Then we may suppose that $u_{n}\to u$ in $L^{q_{1}}(\mathbb {R}^{N})\cap L^{q_{2}}(\mathbb {R}^{N})$ as $n\to\infty$. By the convergence principle, there exist a subsequence $\{u_{n_{k}}\}$ such that $u_{n_{k}}(x)\to u(x)$ as $k\to\infty$ for almost all $x\in\mathbb {R}^{N}$ and a function $u_{0}\in L^{q_{1}}(\mathbb {R}^{N})\cap L^{q_{2}}(\mathbb {R}^{N})$ such that $|u_{n_{k}}(x)|\leq u_{0}(x)$ for all $k\in\mathbb {N}$ and for almost all $x\in\mathbb {R}^{N}$.

First we prove that Ψ is weakly strongly continuous on *X*. Since $F\in C^{1}(\mathbb {R},\mathbb {R})$, we see that $F(u_{n_{k}})\to F(u)$ as $k\to\infty$ for almost all $x\in\mathbb {R}^{N}$ and so $(I_{\alpha}*F(u_{n_{k}}))F(u_{n_{k}})\to(I_{\alpha}*F(u))F(u)$ as $k\to\infty$. From (F2), it follows that

$$\begin{aligned} \int_{\mathbb {R}^{N}} \bigl\vert \bigl(I_{\alpha}*F(u_{n_{k}}) \bigr)F(u_{n_{k}}) \bigr\vert \,dx&\leq\mathcal {M} \int_{\mathbb {R}^{N}}\frac{\sigma}{q_{1}} \bigl\vert u_{n_{k}}(x) \bigr\vert ^{q_{1}}+\frac{\sigma }{q_{2}} \bigl\vert u_{n_{k}}(x) \bigr\vert ^{q_{2}} \,dx \\ &\leq\mathcal{M}\sigma\bigl( \Vert u_{0} \Vert _{L^{q_{1}}(\mathbb {R}^{N})}^{q_{1}}+ \Vert u_{0} \Vert _{L^{q_{2}}(\mathbb {R}^{N})}^{q_{2}}\bigr). \end{aligned}$$

Therefore, the Lebesgue convergence theorem tells us that

$$\int_{\mathbb {R}^{N}} \bigl(I_{\alpha}*F(u_{n_{k}}) \bigr)F(u_{n_{k}}) \,dx \to \int_{\mathbb {R}^{N}} \bigl(I_{\alpha}*F(u)\bigr)F(u) \,dx $$

as $k\to\infty$, which implies $\Psi(u_{n_{k}}) \to\Psi(u)$ as $k\to\infty$. Thus Ψ is weakly strongly continuous on *X*.

Next, we show that $\Psi'$ is weakly strongly continuous on *X*. Since $u_{n_{k}}(x)\to u(x)$ as $k\to \infty$ for almost all $x\in\mathbb {R}^{N}$, $f(u_{n_{k}})\to f(u)$ for almost all $x\in\mathbb {R}^{N}$ as $k\to\infty$. Hence,

$$\bigl(I_{\alpha}*F(u_{n_{k}})\bigr)f(u_{n_{k}}) \rightarrow\bigl(I_{\alpha}*F(u)\bigr)f(u)\quad \text{as } k\to\infty. $$

By (F2) and Hölder’s inequality, we obtain for any $\varphi\in X$

$$\begin{aligned} &\int_{\mathbb {R}^{N}} \bigl\vert \bigl(I_{\alpha}*F(u_{n_{k}}) \bigr)f(u_{n_{k}})\varphi (x) \bigr\vert \,dx \\ &\quad \leq \int_{\mathbb {R}^{N}} \bigl\vert \bigl(I_{\alpha}*F(u_{n_{k}}) \bigr) \bigr\vert \sigma\bigl(|u_{n_{k}}|^{q_{1}-1}+|u_{n_{k}}|^{q_{2}-1} \bigr) \bigl\vert \varphi (x) \bigr\vert \,dx \\ &\quad \leq\mathcal{M}\sigma \int_{\mathbb {R}^{N}}\bigl(|u_{n_{k}}|^{q_{1}-1}+|u_{n_{k}}|^{q_{2}-1} \bigr) \bigl\vert \varphi(x) \bigr\vert \,dx \\ &\quad \leq\mathcal{M}\sigma \Vert u_{n_{k}} \Vert _{L^{q_{1}}(\mathbb {R}^{N})}^{q_{1}-1} \Vert \varphi \Vert _{L^{q_{1}}(\mathbb {R}^{N})}+\mathcal{M}\sigma \Vert u_{n_{k}} \Vert _{L^{q_{2}}(\mathbb {R}^{N})}^{q_{2}-1} \Vert \varphi \Vert _{L^{q_{2}}(\mathbb {R}^{N})} \\ &\quad \leq\mathcal{M}\sigma\bigl( \Vert u_{0} \Vert _{L^{q_{1}}(\mathbb {R}^{N})}^{q_{1}-1} \Vert \varphi \Vert _{X}+ \Vert u_{0} \Vert _{L^{q_{2}}(\mathbb {R}^{N})}^{q_{2}-1} \Vert \varphi \Vert _{X}\bigr). \end{aligned}$$

Combining this with the Lebesgue convergence theorem, we have

$$\begin{aligned} \bigl\Vert \Psi'(u_{n_{k}})-\Psi'(u) \bigr\Vert _{X^{*}}&=\sup_{ \Vert \varphi \Vert _{X}\leq 1} \bigl\vert \bigl\langle \Psi'(u_{n_{k}})-\Psi'(u),\varphi\bigr\rangle \bigr\vert \\ &=\sup_{ \Vert \varphi \Vert _{X}\leq1} \int_{\mathbb {R}^{N}} \bigl\vert \bigl(I_{\alpha}*F(u_{n_{k}}) \bigr)f(u_{n_{k}})\varphi (x)-\bigl(I_{\alpha }*F(u)\bigr)f(u) \varphi(x) \bigr\vert \,dx \\ &\to0 \quad \text{as } k\to\infty. \end{aligned}$$

Therefore, we derive that $\Psi'(u_{n_{k}})\to\Psi'(u)$ in ${X^{*}}$ as $k\to\infty$. This completes the proof. □

### A.2 Proof of Proposition 3.6

Following [36], we give the proof of Proposition 3.6.

#### Proof

Suppose that *u* is nonnegative. For a positive constant *M*, define

$$ v_{M}(x)=\min\bigl\{ u(x),M\bigr\} $$

and choose $v=v_{M}^{kp+1}$ ($k\ge0$) as a test function in (2.1). Then obviously $v\in X\cap L^{\infty}(\mathbb {R}^{N})$ and it follows from (2.1) that

A.1 $$\begin{aligned}& \int_{\mathbb {R}^{N}} \vert \nabla u \vert ^{p-2}\nabla u \cdot\nabla v_{M}^{kp+1} \,dx + \int_{\mathbb {R}^{N}} V(x)|u|^{p-2}u v_{M}^{kp+1} \,dx \\& \quad =\lambda \int_{\mathbb {R}^{N}}\bigl(I_{\alpha}*F(u)\bigr)f(u) v_{M}^{kp+1} \,dx \end{aligned}$$

Due to Lemma 2.1, the left-hand side of (A.1) can be estimated as follows:

A.2 $$\begin{aligned} & \int_{\mathbb {R}^{N}} \vert \nabla u \vert ^{p-2}\nabla u \cdot\nabla v_{M}^{kp+1} \,dx + \int_{\mathbb {R}^{N}} V(x)|u|^{p-2}u v_{M}^{kp+1} \,dx \\ &\quad \ge(kp+1) \int_{\mathbb {R}^{N}}v_{M}^{kp} \vert \nabla v_{M} \vert ^{p} \,dx + \int_{\mathbb {R}^{N}}V(x)v_{M}^{(k+1)p} \,dx \\ &\quad \ge\frac{kp+1}{(k+1)^{p}} \int_{\mathbb {R}^{N}} \bigl\vert \nabla v_{M}^{k+1} \bigr\vert ^{p} \,dx + \frac{1}{(k+1)^{p}} \int_{\mathbb {R}^{N}}V(x) v_{M}^{(k+1)p} \,dx \\ &\quad \ge\frac{1}{C^{p}_{1}(k+1)^{p}} \biggl( \int_{\mathbb {R}^{N}} |v_{M}|^{(k+1)p^{*}} \,dx \biggr)^{\frac{p}{p^{*}}} \end{aligned}$$

for some constant $C_{1}>0$. By using the assumption (F2) and the Hölder inequality, the right-hand side of (A.1) can be formally estimated from above and we obtain

A.3 $$\begin{aligned} &\lambda \int_{\mathbb {R}^{N}}\bigl(I_{\alpha}*F(u)\bigr)f(u) v_{M}^{kp+1} \,dx \\ &\quad \le\mathcal{M}\lambda \int_{\mathbb {R}^{N}}|f(u)||u|^{kp+1} \,dx \\ &\quad \le\mathcal{M}\lambda{\sigma} \biggl( \int_{\mathbb {R}^{N}}|u|^{(k+1)p}|u|^{q_{1}-p} \,dx+ \int_{\mathbb {R}^{N}}|u|^{(k+1)p}|u|^{q_{2}-p} \,dx \biggr) \\ &\quad \le\mathcal{M}\lambda{\sigma} \biggl\{ \biggl( \int_{\mathbb {R}^{N}}|u|^{(k+1)s_{1}} \,dx \biggr)^{\frac{p}{s_{1}}} \biggl( \int_{\mathbb {R}^{N}}|u|^{\frac{s_{1}(q_{1}-p)}{s_{1}-p}} \,dx \biggr)^{\frac {s_{1}-p}{s_{1}}} \\ &\qquad {} + \biggl( \int_{\mathbb {R}^{N}}|u|^{(k+1)s_{2}} \,dx \biggr)^{\frac {p}{s_{2}}} \biggl( \int_{\mathbb {R}^{N}}|u|^{\frac{s_{2}(q_{2}-p)}{s_{2}-p}} \,dx \biggr)^{\frac{s_{2}-p}{s_{2}}} \biggr\} , \end{aligned}$$

where $s_{i}=\frac{pp^{*}}{p^{*}-(q_{i}-p)}$ for $i=1,2$. Obviously $p< s_{i}\le p^{*}$ and $\frac{(q_{i}+1-p)s_{i}}{s_{i}-p}=p^{*}$ for $i=1,2$, and hence (A.3) yields

A.4 $$ \lambda \int_{\mathbb {R}^{N}}\bigl(I_{\alpha}*F(u)\bigr)f(u) v_{M}^{kp+1} \,dx \le C_{2}\lambda \biggl( \int_{\mathbb {R}^{N}}|u|^{p^{*}} \,dx \biggr)^{\frac {s-p}{s}} \biggl( \int_{\mathbb {R}^{N}}|u|^{(k+1)s} \,dx \biggr)^{\frac{p}{s}} $$

for some constant $C_{2}$, where $(q,s)$ is either $(q_{1},s_{1})$ or $(q_{2},s_{2})$. Now it follows from (A.1), (A.2), (A.4), and the Sobolev inequality that there exists a constant $C_{3}>0$ (independent of *M* and $k>0$) such that

$$ \biggl( \int_{\mathbb {R}^{N}} |v_{M}|^{(k+1)p^{*}} \,dx \biggr)^{\frac {p}{p^{*}}}\le C_{3} (k+1)^{p} \biggl( \int_{\mathbb {R}^{N}} |u|^{(k+1)s} \,dx \biggr)^{\frac{p}{s}}, $$

which implies

A.5 $$ \Vert v_{M} \Vert _{L^{(k+1)p^{*}}(\mathbb {R}^{N})} \le C_{3}^{\frac{1}{(k+1)p}} (k+1 )^{\frac{1}{k+1}} \Vert u \Vert _{L^{(k+1)s}(\mathbb {R}^{N})}. $$

Equation (A.5) is a starting point for a bootstrap argument which plays an important role in $L^{\infty}$-estimates. Since $u\in X$ and hence $u\in L^{p^{*}}(\mathbb {R}^{N})$ we can choose $k:=k_{1}$ in (A.5) such that $(k_{1}+1)s=p^{*}$, i.e. $k_{1}=\frac{p^{*}}{s}-1$. Then we have

A.6 $$ \Vert v_{M} \Vert _{L^{(k_{1}+1)p^{*}}(\mathbb {R}^{N})} \le C_{3}^{\frac{1}{(k_{1}+1)p}} (k_{1}+1 )^{\frac{1}{k_{1}+1}} \Vert u \Vert _{L^{(k_{1}+1)s}(\mathbb {R}^{N})}. $$

Due to $u(x)= \lim_{M\rightarrow\infty}v_{M}(x)$ for almost every $x\in\mathbb {R}^{N}$, the Fatou lemma and (A.6) imply

A.7 $$ \Vert u \Vert _{L^{(k_{1}+1)p^{*}}(\mathbb {R}^{N})} \le C_{3}^{\frac{1}{(k_{1}+1)p}} (k_{1}+1 )^{\frac{1}{k_{1}+1}} \Vert u \Vert _{L^{(k_{1}+1)s}(\mathbb {R}^{N})}. $$

Thus, we can choose $k=k_{2}$ in (A.5) such that $(k_{2}+1)s=(k_{1}+1)p^{*}=\frac{(p^{*})^{2}}{s}$ and repeating the same argument we get

$$ \Vert u \Vert _{L^{(k_{2}+1)p^{*}}(\mathbb {R}^{N})} \le C_{3}^{\frac{1}{(k_{2}+1)p}} (k_{2}+1 )^{\frac{1}{k_{2}+1}} \Vert u \Vert _{L^{(k_{2}+1)s}(\mathbb {R}^{N})}. $$

By induction we obtain

A.8 $$ \Vert u \Vert _{L^{(k_{n}+1)p^{*}}(\mathbb {R}^{N})} \le C_{3}^{\frac{1}{(k_{n}+1)p}} (k_{n}+1 )^{\frac{1}{k_{n}+1}} \Vert u \Vert _{L^{(k_{n}+1)s}(\mathbb {R}^{N})} $$

for any $n\in\mathbb {N}$, where $k_{n}+1= (\frac{p^{*}}{s} )^{n}$. It follows from (A.7) and (A.8) that

$$ \Vert u \Vert _{L^{(k_{n}+1)p^{*}}(\mathbb {R}^{N})} \le C_{3}^{\frac{1}{p}\sum_{j=1}^{n}\frac{1}{k_{j}+1}} (k_{1}+1 )^{\frac{1}{k_{1}+1}} (k_{2}+1 )^{\frac {1}{k_{2}+1}}\cdots (k_{n}+1 )^{\frac{1}{k_{n}+1}} \Vert u \Vert _{L^{(k_{1}+1)s}(\mathbb {R}^{N})}. $$

Since $(k_{n}+1 )^{\frac{1}{k_{n}+1}}>1$ and $\lim_{k_{n}\rightarrow \infty} (k_{n}+1 )^{\frac{1}{k_{n}+1}}=1$, there exists $C_{4}>1$ (independent of $k_{n}$) such that

A.9 $$ \Vert u \Vert _{L^{(k_{n}+1)p^{*}}(\mathbb {R}^{N})} \le C_{3}^{\frac{1}{p}\sum_{j=1}^{n}\frac{1}{k_{j}+1}}C_{4} \Vert u \Vert _{L^{(k_{1}+1)s}(\mathbb {R}^{N})}. $$

However, $\sum_{j=1}^{n}\frac{1}{k_{j}+1}=\sum_{j=1}^{n} (\frac{s}{p^{*}} )^{nj}$ and $\frac{s}{p^{*}}<1$. Hence it follows from (A.9) that there exists a constant $C_{5}>0$ such that

A.10 $$ \Vert u \Vert _{L^{r_{n}}(\mathbb {R}^{N})} \le C_{5} \Vert u \Vert _{L^{p^{*}}(\mathbb {R}^{N})}, $$

for $r_{n}=(k_{n}+1)p^{*}\rightarrow\infty$ when $n\to\infty$. Let us assume that $\Vert u \Vert _{L^{\infty}(\mathbb {R}^{N})}>C_{5} \Vert u \Vert _{L^{p^{*}}(\mathbb {R}^{N})}$. Then there exist $\eta>0$ and a set *A* of positive measure in $\mathbb {R}^{N}$ such that $u(x)\ge C_{5} \Vert u \Vert _{L^{p^{*}}(\mathbb {R}^{N})}+\eta$ for $x\in A$. It follows that

$$\begin{aligned} \liminf_{r_{n}\rightarrow\infty} \biggl( \int_{\mathbb {R}^{N}} \bigl\vert u(x) \bigr\vert ^{r_{n}} \,dx \biggr)^{\frac{1}{r_{n}}}&\ge \liminf_{r_{n}\rightarrow \infty} \biggl( \int_{A} \bigl\vert u(x) \bigr\vert ^{r_{n}} \,dx \biggr)^{\frac{1}{r_{n}}} \\ & \ge\liminf_{r_{n}\rightarrow \infty} \bigl(C_{5} \Vert u \Vert _{L^{p^{*}}(\mathbb {R}^{N})}+\eta \bigr) \bigl(\operatorname{meas}(A) \bigr)^{\frac{1}{r_{n}}} \\ &=C_{5} \Vert u \Vert _{L^{p^{*}}(\mathbb {R}^{N})}+\eta, \end{aligned}$$

which contradicts (A.10). Therefore

$$ \Vert u \Vert _{L^{\infty}(\mathbb {R}^{N})} \le C_{5} \Vert u \Vert _{L^{p^{*}}(\mathbb {R}^{N})}\le C_{6} $$

for some constant $C_{6}>0$.

If *u* changes sign, we set

$$u^{+}(x)= \max\bigl\{ u(x), 0\bigr\} \quad \mbox{and} \quad u^{-}(x) = \min\bigl\{ u(x), 0\bigr\} . $$

Then it is clear that $u^{+}\in X$ and $u^{-}\in X$. Proceeding by a similar argument to above, we obtain $u^{+}\in L^{\infty}({\mathbb {R}}^{N})$. Likewise, we get $u^{-}\in L^{\infty}({\mathbb {R}}^{N})$. Therefore $u = u^{+} + u^{-}$ is in $L^{\infty}({\mathbb {R}}^{N})$. This completes the proof. □

## References

[1] Lieb E.H., Loss M. (2001). Analysis.

[2] Fröhlich H. (1937). Theory of electrical breakdown in ionic crystals. Proc. R. Soc. Lond. Ser. A.

[3] Hajaiej H. (2012). Schrödinger systems arising in nonlinear optics and quantum mechanics. Part I. Math. Models Methods Appl. Sci..

[4] Moroz I.M., Penrose R., Tod P. (1998). Spherically-symmetric solutions of the Schrödinger–Newton equations. Class. Quantum Gravity.

[5] Rosenfeld L. (1963). On quantization of fields. Nucl. Phys..

[6] Moroz V., Van Schaftingen J. (2017). A guide to the Choquard equation. J. Fixed Point Theory Appl..

[7] Alves C.O. (2016). Investigating the multiplicity and concentration behaviour of solutions for a quasi-linear Choquard equation via the penalization method. Proc. R. Soc. Edinb., Sect. A.

[8] Alves C.O., Figueiredo G.M., Yang M. (2016). Existence of solutions for a nonlinear Choquard equation with potential vanishing at infinity. Adv. Nonlinear Anal..

[9] Alves C.O., Nóbrega A.B., Yang M. (2016). Multi-bump solutions for Choquard equation with deepening potential well. Calc. Var. Partial Differ. Equ..

[10] Gao F., Yang M. (2017). On nonlocal Choquard equations with Hardy–Littlewood–Sobolev critical exponents. J. Math. Anal. Appl..

[11] Ghimenti M., Van Schaftingen J. (2016). Nodal solutions for the Choquard equation. J. Funct. Anal..

[12] Küpper T., Zhang Z., Xia H. (2003). Multiple positive solutions and bifurcation for an equation related to Choquard’s equation. Proc. Edinb. Math. Soc..

[13] Lieb, E.H.: Existence and uniqueness of the minimizing solution of Choquard’s nonlinear equation. Stud. Appl. Math. **57**(2), 93–105 (1976/77)

[14] Lions P.L. (1980). The Choquard equation and related questions. Nonlinear Anal..

[15] Moroz V., Van Schaftingen J. (2013). Groundstates of nonlinear Choquard equations: existence, qualitative properties and decay asymptotics. J. Funct. Anal..

[16] Moroz V., Van Schaftingen J. (2015). Existence of groundstates for a class of nonlinear Choquard equations. Trans. Am. Math. Soc..

[17] Moroz V., Van Schaftingen J. (2015). Semi-classical states for the Choquard equation. Calc. Var. Partial Differ. Equ..

[18] Yang M., Zhang J., Zhang Y. (2017). Multi-peak solutions for nonlinear Choquard equation with a general nonlinearity. Commun. Pure Appl. Anal..

[19] Alves C.O., Minbo Y. (2014). Multiplicity and concentration of solutions for a quasilinear Choquard equation. J. Math. Phys..

[20] Ambrosetti A., Rabinowitz P.H. (1973). Dual variational methods in critical point theory and applications. J. Funct. Anal..

[21] Lin X., Tang X.H. (2013). Existence of infinitely many solutions for *p*-Laplacian equations in $\mathbb {R}^{N}$. Nonlinear Anal..

[22] Liu S.B. (2010). On ground states of superlinear *p*-Laplacian equations in $\mathbb {R}^{N}$. J. Math. Anal. Appl..

[23] Liu S.B., Li S.J. (2003). Infinitely many solutions for a superlinear elliptic equation. Acta Math. Sin. Chin. Ser..

[24] Miyagaki O.H., Souto M.A.S. (2008). Superlinear problems without Ambrosetti and Rabinowitz growth condition. J. Differ. Equ..

[25] Alves C.O., Liu S.B. (2010). On superlinear $p(x)$-Laplacian equations in $\mathbb {R}^{N}$. Nonlinear Anal..

[26] Bartsch T., Wang Z.-Q. (1995). Existence and multiplicity results for some superlinear elliptic problems on $\mathbb {R}^{N}$. Commun. Partial Differ. Equ..

[27] Le V.K. (2009). On a sub-supersolution method for variational inequalities with Leray–Lions operators in variable exponent spaces. Nonlinear Anal..

[28] Kim Y.-H., Bae J.-H., Lee J. (2017). The existence of infinitely many solutions for nonlinear elliptic equations involving *p*-Laplace type operators in $\mathbb {R}^{N}$. J. Nonlinear Sci. Appl..

[29] Fan X.-L., Han X.-Y. (2004). Existence and multiplicity of solutions for $p(x)$-Laplacian equations in $\mathbb {R}^{N}$. Nonlinear Anal..

[30] Battaglia L., Van Schaftingen J. (2017). Existence of groundstates for a class of nonlinear Choquard equations in the plane. Adv. Nonlinear Stud..

[31] Willem M. (1996). Minimax Theorems.

[32] Drábek P., Kufner A., Nicolosi F. (1997). Quasilinear Elliptic Equations with Degenerations and Singularities.

[33] Lê A. (2006). Eigenvalue problems for the *p*-Laplacian. Nonlinear Anal..

[34] Heinz H.-P. (1987). Free Ljusternik–Schnirelman theory and the bifurcation diagrams of certain singular nonlinear problems. J. Differ. Equ..

[35] Wang Z.-Q. (2001). Nonlinear boundary value problems with concave nonlinearities near the origin. Nonlinear Differ. Equ. Appl..

[36] Drábek P., Milota J. (2013). Methods of Nonlinear Analysis: Applications to Differential Equations.

